# Supplementary material for: Learning curve and surgical outcome of robotic assisted colorectal surgery with ERAS program
Source: Sci Rep. 2022 Nov 29;12:20566. doi: 10.1038/s41598-022-24665-w (PMC9709162; doi:10.1038/s41598-022-24665-w)
Supplement: Supplementary file 1 — Supplementary Information 1. [file 41598_2022_24665_MOESM1_ESM.zip › LSC Raw data-Ñ[▒K/059/OP N_ñw╝╨░O▒Kñσ.pdf]

W82 / W82

臺中榮民總醫院

[redacted]

基本資料

- 室別： 二醫手術室-31室，手術科別： CRS，手術類別： 常規，日期： 2020/01/09
- 進入時間： 1305 ； 麻醉開始時間： 1310 ； Time Out時間： 1445 ； 劃刀時間： 1446 ； 手術結束時間： 1710 送出時間： 1725 ；
- 麻醉方式： GE

  診斷：CA OF RECTUM 10CM FAV

  手術名稱：1.Low anterior resection with colo-anal anastomosis2.Ileostomy3.Laparoscopy4.

  手術局部用藥：無

  術前預防性抗生素：
  - 抗生素名稱一： veterin igm IV /由麻醉護理師給予
  - 時間： 1430
  - 上次簽章: 林昱君

  ，追加預防性抗生素：
- 手術醫師： 蔣鋒帆
- 麻醉醫師： VS 賴慧卿
-
  - 刷手護士A ： 彭馨慧，時間： 1305-1725
  - 流動護士A ： 林昱君，時間： 1305-1700，接替流動一： 吳若綺，時間： 1700-1725

皮膚狀況

A-V Shunt： 有

術前： 不完整

部位(術前)：

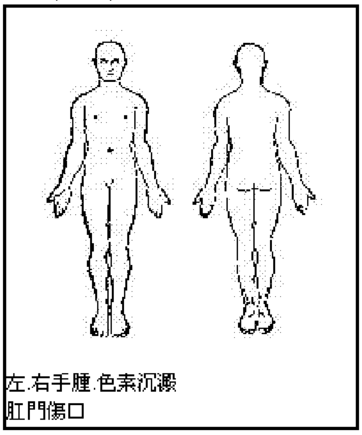

記錄者： 上次簽章: 林昱君

術後： 不完整

部位(術後)：

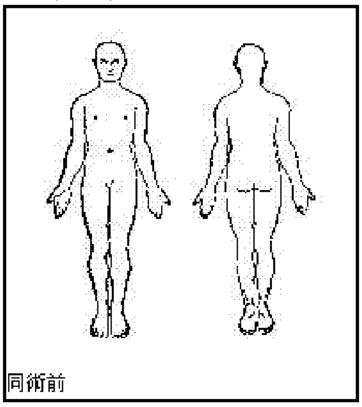

記錄者：

手術準備

手術臥式 單一手術臥式

-
  - 手術臥式： 截石臥式
  - 使用附件： 矽膠枕頭，腳架
  - 皮膚準備： 10%Aq Betadine，Hibiscrub，其它： 2%Chlorhexidine
  - 電燒： 使用電燒: 電極片位置： 其它： 導墊軟墊，記錄者： 上次簽章: 林昱君

止血帶T： 無

植入物

- 植入物名稱/號碼/數量/部位： liga Clip x1 /colon
  End GIA 60/4.1 x2 /colon
  EEA 33mm x1 /colon

, 記錄者： 上次簽章: 林昱君

標本送檢

- 細菌培養 無
- 細胞學 無
- 冰凍切片 無
- 病理標本
  組織名稱一： rectum
  ,
  組織名稱二： IMA root
  ,
  組織名稱三： distal cutend
- 截肢 無

標本交付 標本： 3 袋，標本： 0 盒，標本： 0 瓶，細胞學： 0 管，Culture Tube： 0 管，被交付醫師： CR 江世偉，

- 記錄者： 吳若綺
- 時間： 1720

傷口術後敷料

傷口敷料： 傷口敷料： 無

- 請於圖上標示部位：

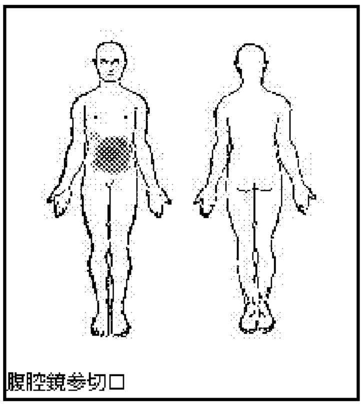

- 傷口敷料： mepore覆蓋固定

導管

導管	天數	導管資訊	插入日期	插入時間	更換日期	拔除日期	拔除時間	拔除原因	預計拔除/更換	預計拔除/更換日期
A-line (1)	1	號碼:22 ; 部位:左腳;	2020/01/09	1400						
NG Tube(1)	1	號碼:16 Fr.; 部位:胃;	2020/01/09	1400						
Penrose Drain(1)	1	號碼:12 mm.; 部位:骨盆腔; 象限:LD;	2020/01/09	1600						

CVC(1)	1	號碼:8 Fr.; 深度:15cm; 部位:jugular (左); 象限:LU;	2020/01/09	1410						
Silicone Foley(1)	15	號碼:16 Fr.; 部位:膀胱;	2019/12/26	1720						

用物計數

- 手術中出血量： 100 c.c.
- 手術中尿量： 200 c.c.
-
    - 其它： 無

用物計數： 多切口同時縫合

- 1.器械： 正確，2.縫針： 正確，3.刀片： 正確，4.紗布： 正確，5.腹部墊： 未使用，6.紗球： 未使用，7.PEANUTS ： 未使用，8.紗條： 未使用，9.絲帶： 未使用，10.針頭： 未使用，11.Vessel loop： 未使用，12.Boots： 未使用，13.Jelco： 未使用，14.棉條： 未使用，
    - 第一次：
        - 刷手護士 ： 彭馨慧
        - 流動護士： 上次簽章: 林昱君
        - 時間： 1420

    ,
    - 第二次：
        - 刷手護士 ： 彭馨慧
        - 流動護士： 上次簽章: 林昱君
        - 時間： 1430

    ,
    - 第三次：
        - 刷手護士 ： 彭馨慧
        - 流動護士： 上次簽章: 林昱君
        - 時間： 1440

備註

備註：NIL

- 記錄者： 吳若綺 上次簽章: 林昱君
- 時間： 1725

填寫護理人員 吳若綺

正式摘要

W82 / W82

doctorRank:B
